# Interleukin-26 activates macrophages and facilitates killing of *Mycobacterium tuberculosis*

**DOI:** 10.1038/s41598-020-73989-y

**Published:** 2020-10-14

**Authors:** Heike C. Hawerkamp, Lasse van Geelen, Jan Korte, Jeremy Di Domizio, Marc Swidergall, Afaque A. Momin, Francisco J. Guzmán-Vega, Stefan T. Arold, Joachim Ernst, Michel Gilliet, Rainer Kalscheuer, Bernhard Homey, Stephan Meller

**Affiliations:** 1 Department of Dermatology, Medical Faculty, Heinrich-Heine-University, Düsseldorf, Germany; 2 Department of Pharmaceutical Biology and Biotechnology, Heinrich-Heine-University, Düsseldorf, Germany; 3 Department of Dermatology, University Hospital CHUV, Lausanne, Switzerland; 4 Department of Biology, Molecular Mycology, Heinrich-Heine-University, Düsseldorf, Germany; 5 Division of Biological and Environmental Sciences and Engineering (BESE), Computational Bioscience Research Center (CBRC), King Abdullah University of Science and Technology (KAUST), Thuwal, Saudi Arabia

**Keywords:** Inflammatory diseases, Tuberculosis

## Abstract

Tuberculosis-causing *Mycobacterium tuberculosis* (Mtb) is transmitted via airborne droplets followed by a primary infection of macrophages and dendritic cells. During the activation of host defence mechanisms also neutrophils and T helper 1 (T_H_1) and T_H_17 cells are recruited to the site of infection. The T_H_17 cell-derived interleukin (IL)-17 in turn induces the cathelicidin LL37 which shows direct antimycobacterial effects. Here, we investigated the role of IL-26, a T_H_1- and T_H_17-associated cytokine that exhibits antimicrobial activity. We found that both IL-26 mRNA and protein are strongly increased in tuberculous lymph nodes. Furthermore, IL-26 is able to directly kill Mtb and decrease the infection rate in macrophages. Binding of IL-26 to lipoarabinomannan might be one important mechanism in extracellular killing of Mtb. Macrophages and dendritic cells respond to IL-26 with secretion of tumor necrosis factor (TNF)-α and chemokines such as CCL20, CXCL2 and CXCL8. In dendritic cells but not in macrophages cytokine induction by IL-26 is partly mediated via Toll like receptor (TLR) 2. Taken together, IL-26 strengthens the defense against Mtb in two ways: firstly, directly due to its antimycobacterial properties and secondly indirectly by activating innate immune mechanisms.

## Introduction

*Mycobacterium tuberculosis* (Mtb)^1–3^ is the causing agent of tuberculosis in humans. Tuberculosis is among the top 10 causes of death worldwide, infecting 10.0 million people and causing 1.5 million deaths in 2018^4^.

A complex interplay between host- and pathogen-specific factors determines the outcome of the infection^5^. Tuberculosis is transmitted via airborne droplets containing Mtb which initially infects, and mainly resides intracellularly in macrophages and also in dendritic cells (DCs)^6^. Mycobacteria enter both cell types via phagocytosis.

The host uses several lines of defense comprising components of the innate as well as the adaptive immune system. Upon infection by Mtb macrophages, DCs, neutrophils, and natural killer (NK) cells detect the pathogen via pattern recognition receptors (PRRs) such as Toll-like receptors (TLRs), Nod-like receptors and C-type lectin receptors^7^. During early infection, macrophages serve as a major site for Mtb replication^8^. During granuloma formation in later stages Mtb may persist in macrophages^8^.

Various endogenous agents such as granulysin, human beta-defensin 2 (hBD2)^9,10^, and LL37 are able to kill Mtb in vitro^11–13^. All of these agents attack Mtb extracellularly as well as inside macrophages. LL37 and hBD2 are secreted by granulocytes as well as epithelial cells and belong to the group of cationic antimicrobial peptides defined by their protein properties and their ability to kill bacteria^14^. Antimicrobial peptides (AMP) protect epithelial surfaces and are part of the innate immune system^14^. Most AMP are cationic peptides sharing the affinity for negatively charged molecules found in the cell envelope of many germs^15^. Briefly, the binding of AMP to a negatively charged microbial compound thins the envelope, finally leading to a pore formation and microbial death by disrupting the cell membrane^14^. Among other proteins and AMPs, LL37 released by neutrophils together with nucleic acids forms neutrophil extracellular traps (NETs). As part of the innate host response, NETs are released upon contact with mycobacteria^16^. Macrophages are able to uptake these NETs containing LL37. Within lysosomal compartments, these AMP-nucleic acid complexes display antimicrobial activity against mycobacteria^17^. Several subtypes of T cells play central role during Mtb infection: Similar to NK cells, cytolytic T lymphocytes and γδ T cells release granulysin and perforin that lyse infected macrophages and inhibit mycobacterial growth^10,18,19^. T_H_1 cells are considered to be the main effector CD4^+^ T cells during tuberculosis infection. Activated T_H_1 cells secrete IFN-γ that induces antimycobacterial activity in macrophages^13,20,21^. Recently, we demonstrated that interleukin-26 (IL-26), a cytokine mainly produced by T_H_1 and T_H_17 cells^22^, but also by innate cells such as NK cells^23^ and macrophages^24^, displays antimicrobial activity^22^. Zhang et al.^25^, showed that IL-26 levels were much higher in tuberculous pleural effusion compared to the corresponding serum. They also demonstrated that IL-26 is induced in T_H_1 and T_H_17 cells upon stimulation with ESAT-6/CFP-10, a Mtb-specific heterodimeric antigen complex^25^. Interestingly, circulating levels of IL-26 decrease after anti-tuberculosis treatment^26^. It was intriguing to assess if IL-26 displays direct antimycobacterial activity.

## Results

### IL26 mRNA is significantly upregulated in both tuberculous lymph nodes and cutaneous sarcoidosis

**Figure 1:**
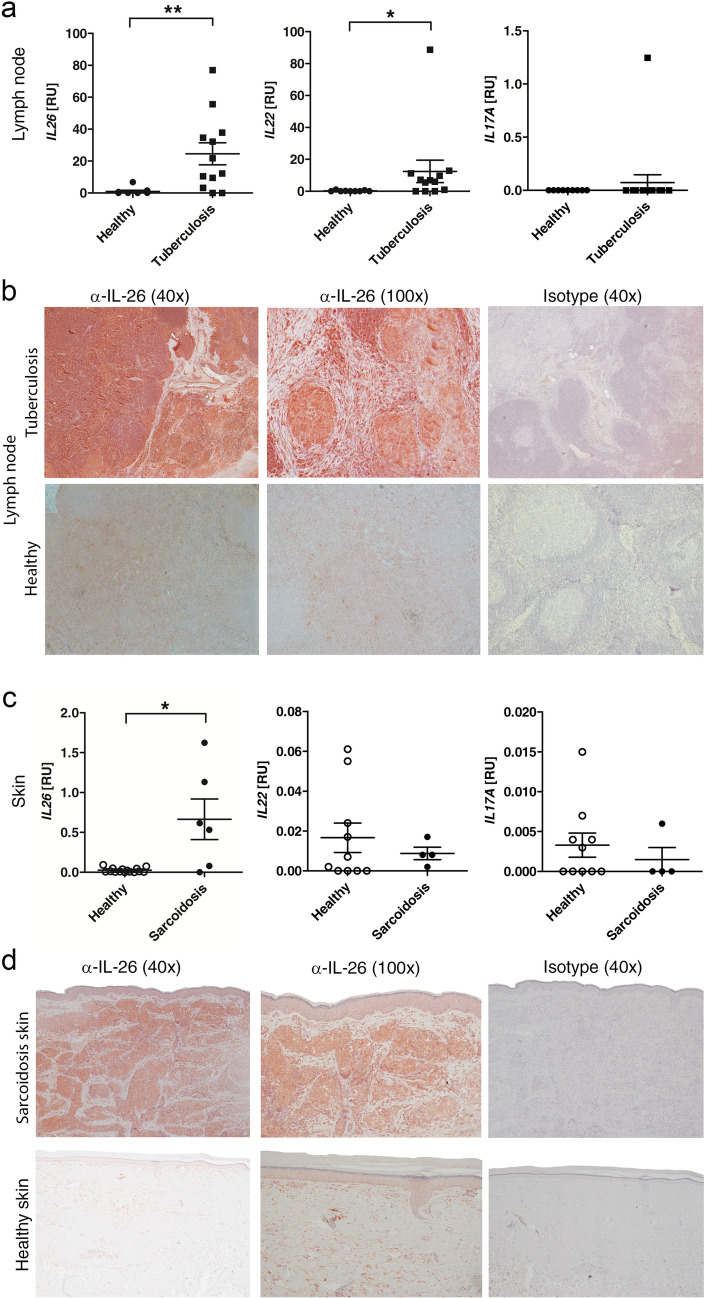
IL-26 is over-expressed in tuberculous lymph nodes and cutaneous sarcoidosis. (**a**, **c**) qPCR analysis of gene expression of *IL26*, *IL22* and *IL17A* in tuberculous LN (**a**, n = 12) compared to healthy control LN (n = 9) in RNA from formalin-fixed paraffin-embedded (FFPE) lymph nodes and sarcoidosis skin punch biopsies (**c**, n = 4–6) compared to healthy skin controls (n = 10). qPCR-values are depicted as relative units compared to 18S RNA expression. Data are presented as single values and mean ± SEM. Mann–Whitney *U* test was used to evaluate significant differences (*p < 0.05, **p < 0.01 and ***p < 0.001). (**b**, **d**) immunohistochemistry with anti-IL-26 and isotype control on FFPE lymph node (LN) sections (10 µm) from one representative tuberculosis patient or healthy control and sections (4 µm) from skin punch biopsies from one representative sarcoidosis patient and one healthy skin donor. Magnification: ×40 for left and right panel, ×100 for middle panel.

A pathological hallmark of tuberculosis is the formation of granulomatous lesions in infected tissues. Comparing lymph nodes (LN) with granulomas from tuberculosis patients to healthy controls by qPCR, we detected a significantly increased gene expression of *IL26* in LN from patients suffering from tuberculosis compared to LN from healthy individuals (Fig. 1a). In addition, *IFNG* (Supplemental Fig. 1a) and *IL22* gene expression (Fig. 1a) is also increased in tuberculosis LN, but the expression of *IL17A* was unaffected (Fig. 1a). In line with the gene expression results IL-26 protein was found to be increased in LN from tuberculosis patients (Fig. 1b upper left and upper middle panel) using immunohistochemistry. In healthy lymph nodes minimal staining for IL-26 was detected (Fig. 1b lower left and lower middle panel).

Another disease with granulomatous appearance similar to tuberculosis is sarcoidosis. Here, we focused on cutaneous sarcoidosis and also report a significant increase in *IL26* gene expression whereas the gene expressions of *IL22* and *IL17A* were not affected (Fig. 1c). Similarly to tuberculous LN, a significantly increased gene expression of IFNG was observed (Supplementary Fig. 1b). Beside the amended gene expression of IL-26, a strongly increased staining for IL-26 protein in the dermal layer of sarcoidosis skin sections was seen (Fig. 1d upper panel). In healthy skin there was only faint IL-26 staining (Fig. 1d lower panel), while strong IL-26 straining was found in psoriatic skin that served as a positive control (Supplementary Fig. 1c)^27^. Here, the staining appears to be mainly associated with infiltrating lymphocytes and proliferating lymphocytes.

### Antimycobacterial activity of IL-26 is supported by binding to lipoarabinomannan (LAM) and via reduced infection rate in macrophages

**Figure 2:**
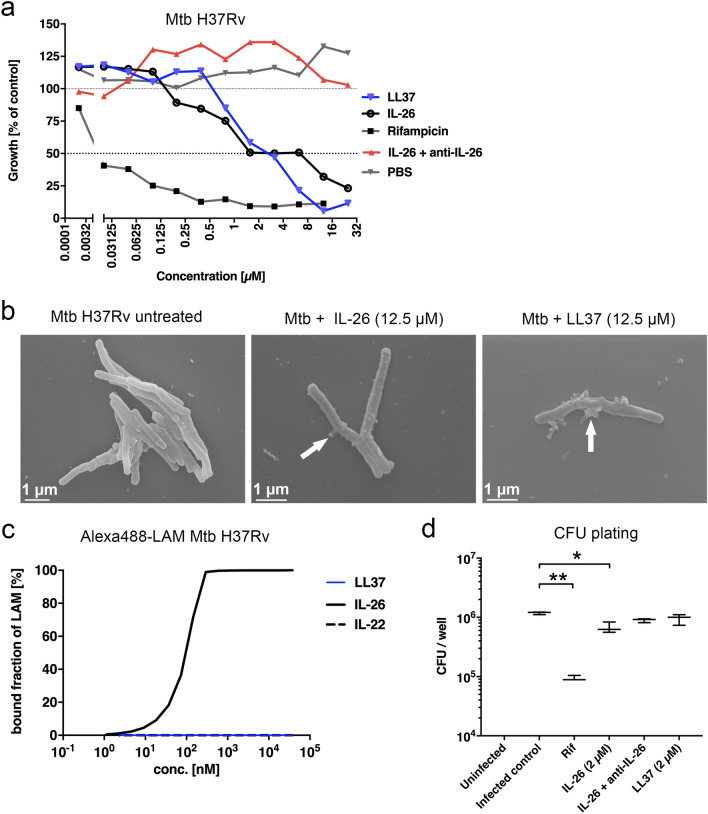
IL-26 displays extracellular antimycobacterial activity against Mtb H37Rv and improves intracellular killing of Mtb H37Rv *pBEN::mCherry* in THP1 macrophages. Growth of MtbH37Rv (**a**) treated with increasing concentrations of IL-26 or LL37 was analyzed using the resazurin assay (n = 3–6). (**b**) Scanning electron microscopy of untreated Mtb (left picture), IL-26-treated Mtb (middle picture) and LL37-treated Mtb (right picture). (**c**) Binding of IL-26 to mycobacterial ManLAM was investigated using microscale thermophoresis (MST). The percentages of bound fractions of LAM from Mtb H37Rv to different IL-26, IL-22 and LL37 concentrations are displayed graphically. (**d**) CFUs from infected THP1 macrophages after treatment with IL-26 (n = 3). Data are presented as mean ± SEM. Statistical analysis was done using Wilcoxon matched pairs signed rank test (* equals p < 0.05, ** equals p < 0.01).

Since we have demonstrated previously that IL-26 is capable of killing some microbes directly^22^, we were wondering if this also applies to mycobacteria. Performing a microbroth dilution assay, we found that IL-26 was indeed able to restrict growth of Mtb (Fig. 2a). IL-26 was similarly effective in inhibiting growth of Mtb as LL37, resulting in a minimal inhibitory concentration (MIC50) of 6 µM compared to 4 µM for LL37 (Fig. 2a). The antimycobacterial effect of IL-26 is completely inhibited in the presence of a blocking anti-IL-26 antibody (Fig. 2a). As expected, the antimycobacterial agent rifampicin showed its strong efficiency also in minimal concentrations (Fig. 2a). To evaluate morphologic changes in mycobacteria in presence of IL-26, we incubated Mtb with 12.5 µM IL-26 or LL37 (positive control) and subsequently prepared the samples for scanning electron microscopy (SEM). The SEM revealed that IL-26 induced “bleb” formation on mycobacteria after 24 h indicative of cell wall damage (Fig. 2b). As most AMPs target components of the mycobacterial cell envelope, we investigated if the major mycobacterial cell-wall glycolipid lipoarabinomannan (LAM) might be targeted by IL-26. Since LAM is known to interact with cationic molecules^28^ we investigated if LAM binds to the cationic IL-26 using microscale thermophoresis and detected a strong affinity of IL-26 to mannosylated (Man) LAM from Mtb H37Rv (Fig. 2c). The calculated dissociation constants were as low as 1 nM for IL-26 to LAM from Mtb H37Rv and also to LAM from *M. smegmatis* (Supplementary Fig. 2a). Moreover, we used computational docking to investigate the molecular basis for the observed interaction between LAM and IL-26 (see “Materials and methods” for details). We performed docking based on the predicted IL-26 structure and on LAM fragments obtained from experimental structure of LAM-protein complexes, namely the oligoarabinofuranosyl tetrasaccharide (complexed to fab; PDB id 3HNT) and Alpha-d-mannose beta-d-mannose *N*-acetyl-d-glucosamine saccharide (complexed to Mycobacterial lipoglycan; PDB id 2GAZ). Without imposing a specific docking site on IL-26, in all high-ranked docking poses both LAM fragments bound to the same surface groove on IL-26 (Supplementary Fig. 2b). The residues surrounding the putative binding site consist of Arg65, Glu155, Asp157, Ser161 forming hydrogen bonds with the ligand, whereas Phe75 and Trp162 involved in hydrophobic interactions (Supplementary Fig. 2c,d). ConSurf analysis^29^ revealed that the binding site residues have intermediate to high conservation (falling in score 5–9 on a scale of 0–9).

The putative LAM binding site is in turn adjacent to a strongly electro-positive region found in the predicted model. The existence of this region was also suggested by Meller et al.^22^. An amphipathic structure, the clustering of cationic charges and the formation of multimers are hallmarks of antimicrobial peptides^14^. These clustered charges have also been shown to form complexes with extracellular DNA^30^. Furthermore, Meller et al.^22^ found IL-26 to be capable of disrupting bacterial membranes by pore formation, and also to bind extracellular DNA from both bacterial and human dying cells.

Mtb primarily invades phagocytic cells such as macrophages or dendritic cells. Within these cells, Mtb may persist and proliferate in modified, host-derived phagosomes^6,31^. A central step in antimycobacterial defense is the phagosome-lysosome fusion leading to the formation of phagolysosomes and subsequent killing of Mtb^31^. Therefore, we investigated if IL-26 is affecting the intracellular killing of Mtb in macrophages using a fluorescent Mtb reporter strain. THP-1 macrophages were infected for 3 h before the addition of IL-26 or LL37 as a control. After 5 days the amount of intracellular mycobacteria was investigated by analysis of colony forming units (CFU). We found a highly significant decrease in presence of rifampicin, but also IL-26 significantly reduced the number of CFUs (Fig. 2d). This implies that IL-26 has intracellular antimycobacterial properties. Preincubation of IL-26 with the blocking anti-IL-26 antibody diminished the intracellular antimycobacterial effect of IL-26 (Fig. 2d).

### *IL-26 induces chemokines as well as cytokines and signals *via* TLR2*

**Figure 3:**
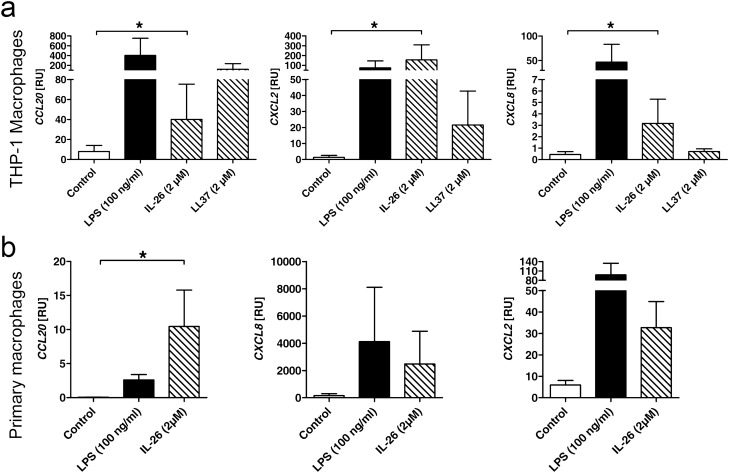
IL-26 induces chemokines in THP1 and primary macrophages. Gene expression of *CCL20*, *CXCL2* and *CXCL8* detected via qPCR in PMA-differentiated THP-1 macrophages (**a**) or primary macrophages (**b**) stimulated with IL-26 and LL37 for 24 h (n = 6). qPCR-values are shown as relative units compared to 18S RNA expression. Data are presented as mean + SEM. Statistical analysis was done using Wilcoxon matched pairs signed rank test (* equals p < 0.05).

We then investigated if IL-26 affects (uninfected) macrophages. Having shown that IL-26 is highly expressed on both gene and protein levels in tuberculosis LN with granulomas—in the latent inactive disease state—packed with different immune cells, we wondered if IL-26 would play a role in cell recruitment via chemotaxis. Treating both THP1 and primary macrophages with IL-26, we found an enhanced gene expression of *CCL20*, *CXCL8* and *CXCL2* (Fig. 3). Comparing this IL-26-induced chemokine induction in THP1 macrophages to treatment with LL37, we found that LL37 induces *CCL20* even stronger than IL-26. For *CXCL2*, LL37 leads to a slight increase, while *CXCL8* is not induced by the presence of LL37 (Fig. 3a).

IL-26 mediates its biological effects through a receptor dimer composed of the IL-20 receptor 1 (IL-20R1) and IL-10 receptor 2^32,33^ or in conjunction with nucleic acids via TLR9^22^. However, macrophages as well as monocyte-derived dendritic cells (moDCs) do not express IL-20R2 and no IL-26-DNA complexes were used in the experiment mentioned above. Seeing the antimicrobial and antimycobacterial effects of IL-26 and thus a link between adaptive and innate immune responses, we wondered if IL-26 might be able to transduce its signals via TLRs. Indeed, TLR2 has been shown to be of importance in the signalling of the antimicrobial human beta-defensin (hBD)-3^34^. Since TLR2, an anionic membrane receptor expressed by leukocytes^35^ that binds exogenous as well as endogenous agents^36^ that may internalized leading to cell activation^37^ and since *CXCL2* expression upon Mtb infection involves TLR2 signaling^38^, we were wondering if IL-26 uses TLR2 to mediate chemokines and cytokine expression in these cells.

**Figure 4:**
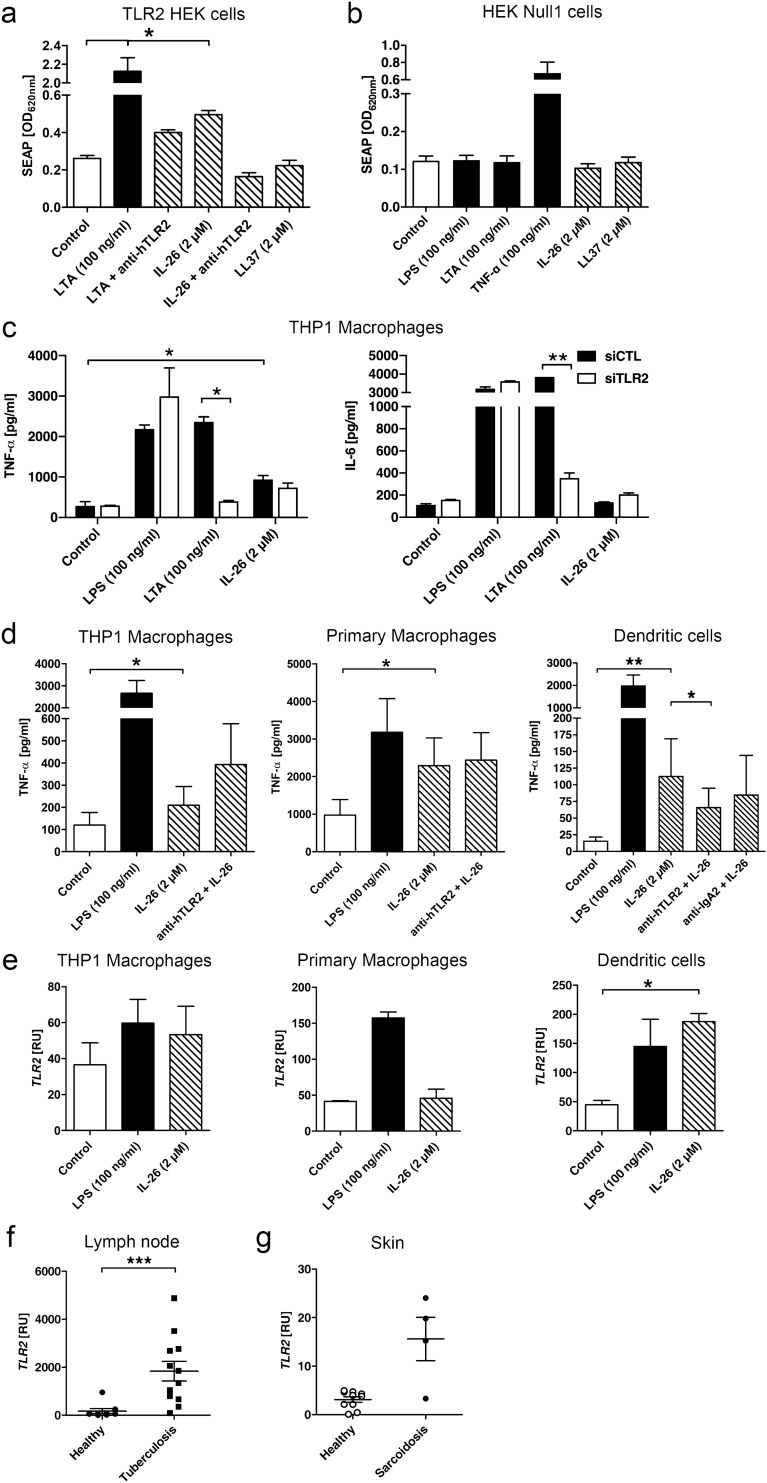
IL-26 exerts effects on moDCs via TLR2. (**a**) Secreted embryonic alkaline phosphatase (SEAP) reporter assay was used to determine if IL-26 is able to signal via TLR2 (n = 4). (**b**) HEK Null1 cells are the parental cell line and are used as controls (n = 3). Statistical analysis was done using Mann Whitney *U* test (* equals p < 0.05). (**c**) TNF-α and IL-6 secretion in THP1 macrophages treated with siTLR2 or control siRNA (siCTL) (n = 2). (**d**) TNF-α secretion by THP1 macrophages, primary macrophages and moDCs treated with IL-26 or pretreated with anti-TLR2-antibody for 45 min before the addition of IL-26 for another 24 h and measured via ELISA (n = 6). (**e**) *TLR2* gene expression after IL-26 stimulation in THP1 macrophages, primary macrophages and DCs (n = 5), or comparing tuberculosis (**f**) or sarcoidosis (**g**) to their respective healthy control. Data are presented as mean + SEM or a single dot is representing one independent sample. Statistical analysis was done using Wilcoxon matched pairs signed rank test (* equals p < 0.05) or Mann–Whitney *U* test for the diseases (*** equals p < 0.001).

In fact, gain-of-function experiments using a TLR2 reporter gene assay confirmed that IL-26 induces NF-κB in TLR2-transfected HEK293 cells (Fig. 4a). The presence of an anti-TLR2-antibody completely blocked the effects of IL-26. Additionally, stimulating the TLR2-transfected HEK293 cells with LL37 revealed no response (Fig. 4a). When stimulating the parental control cell line to the TLR2-transfected HEK cells, the HEK Null1 cells, with IL-26 or LL37, no SEAP secretion is observed (Fig. 4b). The HEK Null1 cells also did not respond to LPS nor LTA, but strongly to 100 ng/mL TNF-α. Next, we compared TLR2 knockdown THP1 macrophages to the standard THP1 macrophages in terms of their TNF-α and IL-6 secretion as response to IL-26 treatment (Fig. 4c). Successful TLR2 knockdown using siRNA in THP1 macrophages was confirmed via flow cytometry (Supplementary Fig. 3a,b), qPCR (Supplementary Fig. 3c) and confocal microscopy (Supplementary Fig. 3d). TLR2 knockdown did not interfere with LPS-induced TNF-α and IL-6 secretion, but treatment with LTA did not lead to TNF-α and IL-6 secretion (Fig. 4c). IL-26 significantly increased TNF-α secretion but had no effect on IL-6 in normal THP1 macrophages. TLR2 knockdown slightly reduced TNF-α secretion after IL-26 treatment as compared to TLR2-competent macrophages, while IL-6 secretion seemed to be enhanced in IL-26-treated TLR2 knockdown macrophages compared to control macrophages (Fig. 4c). Regarding chemokine expressions, there seems to be an impaired induction of *CXCL1* and *CXCL8* in TLR2 knockdown macrophages as compared to controls (Supplementary Fig. 3e).

To get closer to the nature of tuberculosis with involvement of different cell types including antigen-presenting cells such as DCs, we simulated a TLR2 inhibition using a blocking anti-TLR2-antibody. Here, we compared anti-TLR2 antibody treatment in THP1 macrophages, primary macrophages and finally monocyte derived DCs (Fig. 4d). The presence of IL-26 led to a significantly increased TNF-α secretion in all three cell types with the strongest effect in moDCs. IL-26 dependent induction of TNF-α secretion was reduced by adding an inhibitory anti-TLR2 antibody in moDCs (Fig. 4d). This inhibition in moDCs is not seen when IL-26 is co-incubated with an anti-IgA2 control antibody (Fig. 4d). In contrast, an inhibitory anti-TLR2 antibody did not affect the TNF-α in THP1 and primary macrophages (Fig. 4d). We wondered if this different reaction to TLR2 blockade or knockout might be due to variations in *TLR2* gene expressions in these cell types. Indeed, baseline *TLR2* gene expression in untreated THP1 and primary macrophages as well as moDCs is roughly identical (Fig. 4e). Interestingly, IL-26 significantly induced the gene expression of *TLR2* in moDCs but not in THP1 nor primary macrophages (Fig. 4e). Taken together, IL-26 signalling via TLR2 seems to be a cell dependent mechanism. A strongly increased *TLR2* gene expression was found in lymph node of patients suffering from tuberculosis compared to healthy subjects (Fig. 4f). In skin biopsies from sarcoidosis patients a strong trend (p = 0.0539) towards increased *TLR2* expression was observed (Fig. 4g).

**Figure 5:**
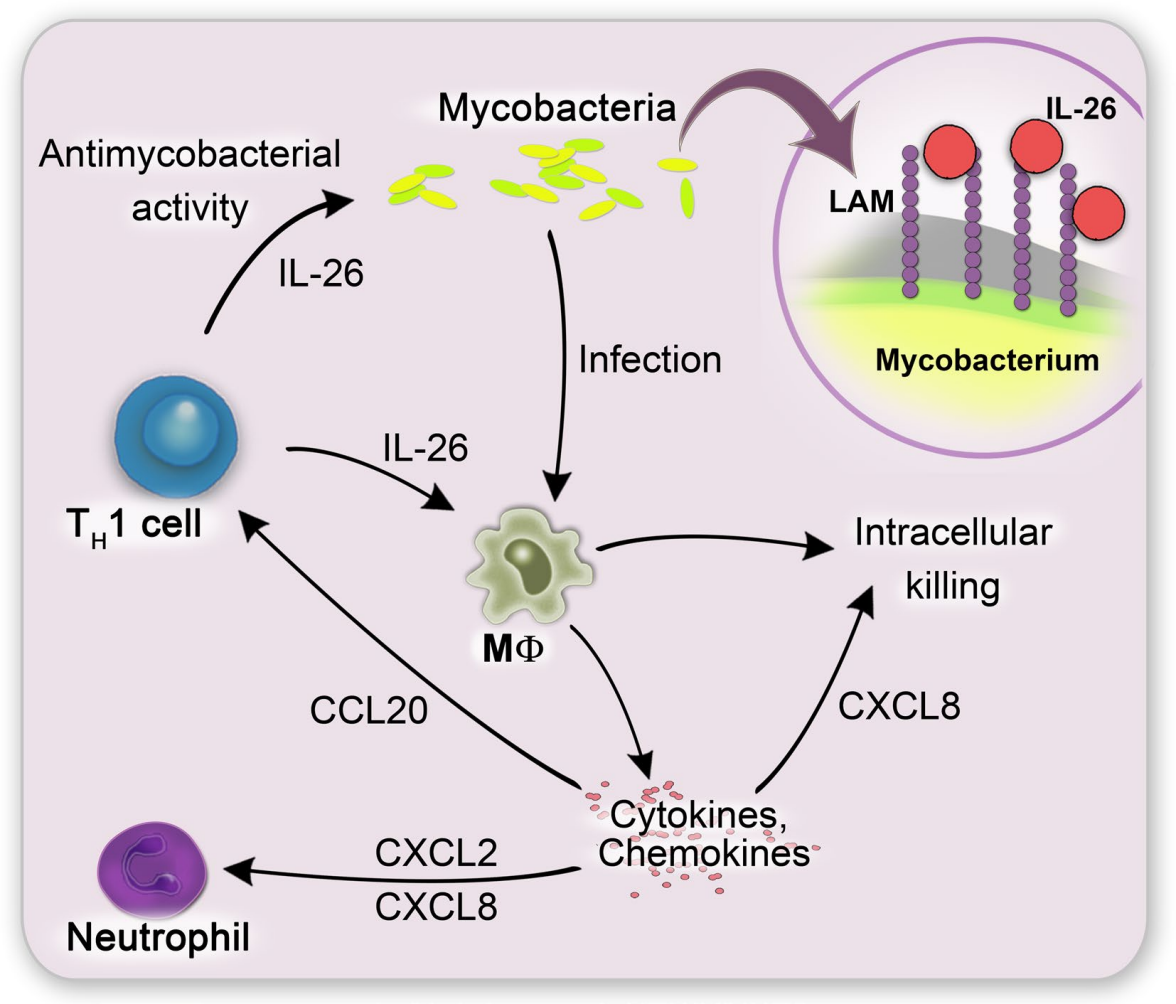
Proposed summarizing model. T_H_1 cell-derived IL-26 is increased expressed in lymph node in tuberculosis patients. IL-26 shows direct antimycobacterial activity. This activity which is possible mediated via binding of IL-26 to mycobacterial surface molecule LAM. Besides direct activity against mycobacteria, IL-26 also improves intracellular killing of mycobacteria in THP1 macrophages. Furthermore, IL-26 induces cytokines such as TNF-α and chemokines such as CCL20, CXCL8 and CXCL2 in macrophages. CCL20 leads then to the further recruitment of T cells to the side of infection and CXCL8 as well as CXCL2 are attracting neutrophils.

We report that IL-26 is able to kill mycobacteria directly (Fig. 5). This killing might be supported through binding to LAM on mycobacterial surface. Furthermore, we showed that the presence of IL-26 improves the intracellular killing of Mtb within macrophages. The increased expression of CCL20, CXCL2, CXCL8 and TNF-α of uninfected macrophages in response to IL-26 may lead to the attraction of T_H_17 cells and neutrophils to inflammation sites (Fig. 5).

## Discussion

We report here that both IL-26 gene and protein expression are significantly increased in tuberculosis and sarcoidosis. The detection of IL-26 in granulomatous lesions of tuberculosis is surprising since LL37, an antimicrobial peptide with great impact on tuberculosis infection^11,12^, was reported to be mostly absent in these chronic lesions of tuberculosis^39^. Among others, LL37 expression is induced by the T_H_17 cytokine IL-17^40^, which was almost undetectable in our cohort. T_H_17 cells also release IL-26 and IL-22. Since IL-26 and IL-22 can also be secreted by other tuberculosis-associated lymphocyte subsets than T_H_17 such as T_H_1 cells or by NK cells^41,42^, our findings point to T_H_1 cells or NK cells as cellular source of IL-26 in granulomatous lesions of tuberculosis.

IL-26 is capable of inhibiting Mtb and creating blebs on the mycobacterial cell surface. Antimicrobial activity against mycobacteria has been shown recently where IL-26 had killing effects on *M. leprae* and the attenuated *M. tuberculosis* strain H37Ra^43^. Comparing the data from the attenuated strain to our data with the virulent Mtb H37Rv, it seems as if Mtb H37Rv is more resistant to IL-26^43^. Treating other microbes with IL-26 has revealed this antimicrobial phenomenon previously as well^22^. Similar surface lesions on Mtb are caused by granulysin, a protein released by cytolytic T cells during antimycobacterial responses^10^. The cell envelope of mycobacteria contains several pathogen-associated molecular patterns (PAMPs)^44^. These PAMPs are recognized by macrophages and DCs through PRRs such as TLRs^44^. The PAMP LAM is a key component of the mycobacterial cell surface and an important virulence factor that is thought to bind TLR2^44,45^. Since LAM interacts with cationic molecules^28^ we tested its binding to cationic IL-26 and found a strong affinity. Our computational modelling analysis suggests that IL-26 possesses a region that is capable of binding to oligosaccharides. Given the large size of LAM, a multimer of several IL-26 molecules might bind collaboratively to one LAM molecule, explaining the high affinity. Considering LL37, it binds with similar affinity as IL-26 to LAM from *M. smegmatis* but it basically does not bind to LAM from Mtb. LAM from Mtb is decorated with terminal mannose residues resulting in Man-LAM whereas LAM from *M. smegmatis* lacks this mannose cap. Instead, *M. smegmatis* LAM is terminated by phosphoinositol, yielding PI-LAM^46^. The additional negative charge in PI-LAM vs. Man-LAM might explain the differential binding of the cationic peptide LL37. Taken together, LAM represents one potential target of the mycobacterial cell wall that—among others—enables IL-26 to attack Mtb extracellularly.

Tuberculosis is characterized by a primary infection of macrophages. We found that the presence of IL-26 improves macrophage survival by decreasing the infection rate of Mtb. This result is in line with a study from Dang et al. showing that IL-26 has similar effects in macrophages infected with M. leprae, Mtb H37Ra or *S. aureus*^43^. Furthermore, uninfected macrophages respond to IL-26 with increased TNF-α secretion and increased expression of the chemokines *CCL20*, *CXCL2* and *CXCL8*. Importantly, TNF-α is a key molecule in tuberculosis defence and an important therapeutical target in sarcoidosis released by monocytes after activation via TLR2^47^. TNF-α induces autophagy and is crucial for the formation of well-organized granulomas. Inhibition of this cytokine goes along with deterioration or reactivation of tuberculosis, absence of TNF-α is associated with fatal tuberculosis progression^48^. Furthermore, the expression of CCL20, CXCL8 and CXCL2 has been implicated in the pathogenesis of tuberculosis. CCL20 is highly upregulated in Mtb-infected macrophages. This may help to recruit more DCs to the site of inflammation^49^. However, the exact role of CCL20 in tuberculosis remains to be clarified. CXCL8 was reported to bind to the Mtb cell surface to increase the potency of leukocytes to phagocytose the germ. The same group suggested that CXCL8, well known as neutrophil attracting chemokine, may serve as a major chemokine for recruiting CD3^+^, CD4^+^, and CD8^+^ T cells^50^. Recent studies indicate that CXCL2 mediates the induction of beta-defensins as well as it is involved Mtb-dependent IL-1β production^38,51^.

As IL-26 acts on the interface between innate and adaptive immunity, we hypothesized that a TLR might act as its receptor on immune cells such as macrophages or DCs. Knowing that TLR2 signaling is involved in CXCL2 expression^38^ and additionally hBD3 signaling on antigen-presenting cells depends on TLR2^34^, we report that TLR2 serves as a putative receptor for IL-26 in a cell type dependent manner. Previous studies have demonstrated that activation of TLR2 leads to the induction of antimicrobial responses including the expression of LL37 and intracellular killing of Mtb via the upregulation of vitamin D receptor and vitamin D-1-hydroxylase genes^12^. An induction of TLR2 expression in presence of a ligand has been reported for LPS^52^. To date, it was known that the activation of TLR2 is mediated by bacterial components^52,53^. Interestingly, the antimycobacterial T cell protein granulysin exerts its effects on antigen-presenting cells via TLR4^54^. IL-26 does not use TLR4 signalling (data not shown). Here, we demonstrated that a T cell-derived cytokine is able to activate TLR2 and this way it may enhance innate immune responses.

In the context of sarcoidosis these results provide new puzzle pieces of the largely unknown pathogenesis of this disease suggesting a role of IL-26 in the activation of moDCs via TLR2 followed by the secretion of TNF-α, an important pathological player and therapeutical target in sarcoidosis.

To conclude, we report that IL-26 is able to kill mycobacteria directly. This killing might be mediated through binding to LAM on mycobacterial surface. Furthermore, we showed that the presence of IL-26 improves the intracellular killing of Mtb within macrophages. Macrophages (uninfected) also respond to IL-26 with increased expression of TNF-α as well as CCL20, CXCL2 and CXCL8. These may lead to the attraction of T_H_1 cells^55^ and neutrophils to inflammation sites. CXCL8 has additionally been shown to enhance Mtb killing within macrophages^56^. The increased gene expression of CXCL8 might therefore provide a mechanism how IL-26 enhances intracellular Mtb killing. It was further demonstrated that secreted CXCL8 binds to Mtb and increases phagocytosis of the mycobacteria^50^.

## Material and methods

### Study approval

Formalin-fixed paraffin-embedded (FFPE) sections (10 µm) from lymph nodes from healthy subjects (n = 9, mean age: 51.6 years) and patients with tuberculosis (n = 12, mean age: 29.8 years) were provided by the Institute of Pathology, University Hospital Düsseldorf. Skin punch biopsies (sections: 4 µm) from sarcoidosis (n = 4–5, mean age: 64 years) and healthy skin (n = 10, mean age: 78 years) were taken after informed consent. Buffy coats from healthy donors were obtained from the Institute of Hemostasis and Transfusion Medicine, University Hospital Düsseldorf. The study was performed according to the Declaration of Helsinki and approved by the Ethics Committee, Medical Faculty of the Heinrich-Heine-University Düsseldorf (no: 4028).

### Immunohistochemistry

FFPE sections were incubated at 60 °C overnight and subsequently washed in a decreasing ethanol series. The sections were incubated for 20 min with demasking solution (pH 9; Dako Target Retrieval), washed in PBS and subsequently incubated in H_2_O_2_ for 10 min. After washing, the sections were incubated with 10% human serum and streptavidin (Vector Laboratories, USA) for 30 min at room temperature (RT). The primary antibodies, anti-IL-26 (Clone: 197505 unconjugated, R&D Systems, USA) and mouse IgG2b isotype (BD Biosciences, USA), were diluted in 1% human serum/PBS and biotin (Vector Laboratories, USA), adjusted to 2 µg/mL and incubated overnight at 4 °C. After washing, the sections were incubated with 10% horse serum for 15 min at RT. Thereafter the secondary antibody (biotinylated anti-mouse IgG; VectaStain) was applied for 30 min at RT. The sections were then incubated with streptavidin horseradish peroxidase (HRP, Dako Agilent Technologies, USA) for 45 min at RT followed by incubation with the AEC 2-Components Kit (DCS Chromokine, Germany) at RT and counterstaining with hematoxylin.

### RNA isolation

RNA Isolation from skin biopsies was performed using Trizol separation, followed by the RNeasy Mini Kit (Qiagen, Venlo, Netherlands). RNA from cultured cells was isolated using the RNeasy Mini Kit. The RNeasy FFPE Kit (Qiagen) was used for FFPE sections.

### Reverse transcriptase PCR

Four µg of RNA were added to 1.5 µL first strand buffer (Invitrogen), 1 µL RNasin^®^ Plus (Promega), 1 µL DNase I recombinant (Roche) and 2.5 µL H_2_O. After the first incubation (20 min at 37 °C, 10 min at 70 °C), 1 µL oligo dT (Invitrogen), 0.4 µL random primer (Promega) and 2.6 µL H_2_O were added and incubated for 10 min at 70 °C. Finally, 4.5 µL first strand buffer, 1.5 µL dNTP Mix (Bioline), 1 µL DTT (Invitrogen), 0.5 µL RNasin^®^ Plus, 1 µL SuperScript^®^ II Reverse Transcriptase (Invitrogen) and 1.5 µL H_2_O were added to each sample. The samples were finally incubated for 50 min at 42 °C and then 10 min at 70 °C.

### Quantitative (q)-PCR

qPCR analysis was performed on a QuantStudio 6 Flex (Applied Biosystems). Briefly, the primer mix for the ribosomal 18 S control gene contained 12.5 µL TaqMan Master Mix (Applied Biosystems), 0.15 µL Ribosomal Probe (life technologies), 0.15 µL Forward Primer (life technologies), 0.15 µL Reverse Primer (life technologies) and 2.05 µL H_2_O per sample. Ten µL of cDNA (25 ng) were analyzed. For TaqMan^®^ Assays (Applied Biosystems; IL17A (Hs00174383_m1), IL22 (Hs00220944_m1), IL26 (Hs00218189_m1), CCL20 (Hs00171125_m1), CXCL2 (Hs00601975_m1) and CXCL8 (Hs00174103_m1)), 10 µL TaqMan Master Mix and 1 µL primer mix was added. For TLR2 primers (forward: cgttctctcaggtgactgctc, reverse: cctttggatcctgcttgc), 12.5 µL SYBR Green Master Mix and 2.5 µL of a 2 µM primer mix were used.

### Mycobacteria

*Mycobacterium tuberculosis* H37Rv (Mtb), (Albert Einstein College of Medicine, Bronx, USA) and *M. bovis* Bacille de Calmette et Guérin (BCG) (Institute Pasteur) were cultured in Difco™ Middlebrook 7H9 Broth (BD, Franklin Lakes, US) supplemented with 10% ADS (5%, w/v bovine serum albumin fraction V; 2%, w/v glucose; 0.85%, w/v sodium chloride), 0.5% (v/v) glycerol, and 0.05% (v/v) tyloxapol at 37 °C.

### Microbroth dilution assay

Mtb and *M. bovis* BCG were diluted to a final concentration of 1 × 10^6^ CFU/mL in RPMI 1640 (incl. NaHCO_3_ and l-Glutamine; Gibco) diluted 1:4 in distilled water. Now, 50 µL of diluted RPMI medium was transferred into all wells of the polypropylene 96-well-plate except the first column. In each well of the first column, the reagents to test [e.g. IL-26 (R&D Systems), LL37 (InvivoGen, USA), rifampicin (Sigma-Aldrich, Munich, Germany)] as well as controls were diluted. Controls included diluted RPMI 1640 without any added bacteria or bacteria in diluted RPMI 1640 as growth controls. Furthermore, to account for possible effects of the solvents/diluents, matching volumes of PBS (for IL-26, anti-IL-26 and LL37) and DMSO (for rifampicin) were added as controls as well. Thereafter, a twofold serial dilution was performed. For experiments involving the blocking anti-IL26-antibody^22^, five dilutions of IL-26 (starting with 25 µM) were prepared in Eppendorf tubes and an equal amount of 10 µg/mL anti-IL-26 was added to each tube. This mixture was then incubated for 1 h at room temperature before transfer into the 96-well-plate. The mycobacteria were added in a volume of 50 µL. The 96-well-plate was incubated for 5 days at 37 °C, before an aliquot was obtained to determine colony forming units (CFU). Subsequently, 10 µL resazurin (Sigma-Aldrich) was added to 96-well-plate and measured after overnight incubation at RT using a plate reader (Ex/Em: 540/590 nm; TECAN Infinite^®^ 200; Männedorf, Switzerland).

### Scanning electron microscopy

Mycobacteria (5 × 10^5^ CFU/mL in diluted RPMI medium) were transferred into a 24-well-plate containing poly-l-lysine-coated round cover glasses. After addition of IL-26 or LL37, the mycobacteria were incubated for 24 h. The mycobacteria were fixed with 2.5% glutaraldehyde and 4% paraformaldehyde (PFA). After refrigerated overnight incubation, the cover glasses were first washed with PBS followed by a serial dehydration starting with 50% ethanol (EtOH) and increasing to pure EtOH. Thereafter the cover glasses were washed twice with 100% acetone. The dehydrated samples were then subjected to critical point drying (CPD). The dried cover glasses were sputtered with gold using a Manual Sputter Coater (Agar Scientific, Essex, UK). Scanning electron microscopy (SEM) was performed using a Leo 1430 VP (Zeiss, Jena, Germany).

### Microscale thermophoresis

Purified lipoarabinomannan (LAM) from Mtb H37Rv and *Mycobacterium smegmatis* were obtained through BEI Resources, NIAID, NIH, USA. LAMs were labeled with the Alexa Fluor 488^®^ Microscale Protein Labeling Kit (life technologies). A constant amount of Alexa488-labeled LAM (200 nM) was incubated for 15 min at 37 °C with serially diluted concentrations of IL-26, LL37 or IL-22 (R&D Systems) in microscale thermophoresis (MST) binding buffer (50 mM Tris and 0.05% Tween 20). Ten μL of the samles were loaded into standard Monolith NT Capillaries and analyzed on a NanoTemper Monolith NT.115 apparatus.

### Computational modeling

The three-dimensional Interleukin-26 (IL-26) structure was modeled using the I-Tasser^57^ protein structure prediction server. The model was built for human IL-26 residues 22–171 (the first 21 residues are predicted to be a signal peptide; Uniprot entry Q9NPH9), based on different threading templates, including structures of IL-10, IL-19, IL-22 and IL-24 which have a sequence identity to IL-26 of 23–28%. The best I-Tasser model for IL-26_22–171_ achieved a C-score of − 0.17 [the C-score ranges from − 5 (poor) to + 2 (excellent)], and a TM-score of 0.69 ± 0.12 (a TM-score of > 0.5 indicates a model of correct topology). The IL-26 model was used for molecular docking with a linear, terminal oligoarabinofuranosyl tetrasaccharide from lipoarabinomannan (LAM) obtained from its experimental structure with fab (PDB id 3HNT) and alpha-d-mannose beta-d-mannose *N*-acetyl-d-glucosamine saccharide from its experimental structure with Mycobacterial lipoglycan (PDB id 2GAZ).

Flexible docking was performed using AutoDock 4.2 with specific coordinate file types for both protein and ligand, termed PDBQT files. The files were prepared using the AutoDock Tools 4.2 user interface^58,59^. Water molecules were removed, polar hydrogens were added, and the structures were saved as PDBQT. The flexible ligand was prepared by assigning the atom types, analyzing hydrogen bond acceptors and donors with the aromatic and aliphatic carbon atoms. The root was defined for the torsion tree from which the rotatable bonds emanate and define the flexibility of the ligand. Finally, the rotatable bonds and torsion angles were assigned and the files were saved as PDBQT^60^. The grid parameters were set in accordance to the binding pocket^61^. Docking was performed using Lamarckian genetic algorithm (LGA) with population size of 150 individuals, 2.5 million energy evaluations, maximum of 27,000 generations, number of top individuals to automatically survive to next generation of 1, mutation rate of 0.02, crossover rate of 0.8, 10 docking runs, and random initial positions and conformations. The probability of performing local search on an individual in the population was set to 0.06 to get optimal results^62,63^. The files were further analyzed using the PyMOL program (pymol.org).

### THP-1 macrophages

THP-1 monocytes (Deutsche Sammlung von Mikroorganismen und Zellkulturen GMBH) were cultured in RPMI 1640 medium (Gibco) supplemented with 10% FCS (without antibiotics). The cells (1 × 10^6^ cells/mL) were used at 100 µL per well in a 96-well-plate. Adherent macrophages were generated by the addition of 50 nM phorbol 12-myristate 13-acetate (PMA, Sigma) overnight.

### Intracellular killing assay with THP-1 macrophages

THP-1 macrophages were washed twice with PBS and then rested for 3 h in RPMI medium. The cells were then infected with a *Mycobacterium tuberculosis* H37Rv reporter strain [Mtb *pBEN::mCherry* (Hsp60)], that expresses mCherry under control of a promotor of heat shock protein 60 (Hsp60). The THP-1 macrophages were infected with 3 × 10^5^ mycobacteria (MOI = 3) in a final volume of 20 µL for 3 h. After 2 h of incubation, 10 µg/mL anti-hTLR2-antibody or anti-IgA2 control antibody (Both: InvivoGen) were added to the respective wells for the remaining 1 h incubation time. After another washing step, fresh medium including gentamycin (5 µg/mL, Sigma-Aldrich) was added to all wells. At this point the reagents such as IL-26 (final: 2 µM), LL37 (final: 10 µM), rifampicin (final: 10 µM) and controls were added. As untreated control served uninfected THP1 macrophages in RPMI, and as treated control served infected THP1 macrophages in RPMI. DMSO as solvent of rifampicin and PBS (for IL-26 and LL37) were used to exclude effects due solvents or diluents. The THP-1 macrophages were incubated at 37 °C for 5 days, before CFU plating was performed. In order to determine CFUs, THP1 macrophages were lysed using 0.05% SDS and serial dilutions were spread on agar plates.

### Stimulation of uninfected THP-1 macrophages

Uninfected THP-1 macrophages (1 × 10^6^ cells/mL) were cultured in RPMI 1640 medium (control) or stimulated with LPS (100 ng/mL, Sigma-Aldrich), LL37 (10 µM) and IL-26 (2 µM) for 24 h at 37 °C. The supernatant was for ELISA and the cell pellets for gene expression analysis.

### Secreted embryonic alkaline phosphatase (SEAP) reporter assay

HEK-Blue™ hTLR2 cells (InvivoGen) are human embryonic kidney cells (HEK293 cells) specially engineered to co-express human TLR2 and secreted embryonic alkaline phosphatase (SEAP). Furthermore, a CD14 co-receptor gene was introduced into these cells to enhance TLR2 response. The SEAP reporter gene is under the control of the NF-κB/AP-1 promoter. In those HEK-Blue™ hTLR2 cells stimulation with a TLR2 ligand will lead to SEAP secretion. The secreted SEAP can then be detected in real time using HEKBlue™ Detection, a specific cell culture medium. This medium contains a substrate that upon hydrolysis by SEAP changes colour from pink to purple/blue. The control cells are the HEK-Blue™ Null1 cells (InvivoGen), which are the parental cell line and exhibit an endogenous expression of TLR3, TLR5 and NOD1, but are deficient for all other TLRs. The SEAP reporter assay for both cell lines was performed according to manufacturer’s instructions. Briefly, 20 µL of each reagent or control were added per well in a flat-bottom 96-well-plate. In different experiments IL-26 (2 µM) or IL-26 (2 µM) in combination with 10 µg/mL anti-hTLR2 (InvivoGen) have been used. Anti-hTLR2-antibody was added 45 min prior to addition of IL-26. As positive controls LTA (100 ng/mL; InvivoGen) was used in HEK-Blue™ hTLR2 cells, while TNFα (100 ng/mL; R&D Systems) was used in HEK-Blue™ Null1 cells. HEK-Blue™ hTLR2 cells were adjusted to a concentration of 50,000 cells per well in HEKBlue™ Detection medium and incubated for 24 h. SEAP secretion as indicated by a change of cell culture medium colour from pink to purple or blue was measured at 620 nm.

### Generation of TLR2 knockdown THP-1 cells

THP-1 cells (ECACC, 88081201) were cultured in RPMI (incl. 2 mM Glutamine and 10% FBS) and were differentiated into macrophages by adding 150 nM PMA for 24 h followed by a 4-day culture. THP-1 macrophages were washed and cultured in antibiotic-free medium 24 h before siRNA transfection according to the manufacturer’s protocol (Santa Cruz Biotechnology). Basically, 2 × 10^5^ macrophages in a 6-well plate were washed with siRNA Transfection Medium and 1 mL of Transfection Medium containing 80 pmols of control siRNA (siCTL) or siRNA against human TLR2 (siTLR2) and 8 μL of Transfection Reagent was added to the cells. The cells were incubated for 6 h at 37 °C before 1 mL of culture medium was added. Forty-eight hours later, cells were assessed for TLR2 expression by flow cytometry.

### Flow cytometry

Cells (5 × 10^5^ cells) were washed and re-suspended in 100 μL of PBS (incl. 2% FBS and 2 mM EDTA) containing 20 μL of FcR Blocking Reagent (Miltenyi) for 10 min at 4 °C. Cells were then stained with anti-human CD33-FITC (1/200, eBioscience, HIM3-4) and anti-human TLR2-PE (1/50, BioLegend, clone QA16A01) antibodies for 20 min at 4 °C. Cells were then washed twice and acquired on a FACSCalibur (BD) and analyzed using FlowJo software (Tree Star, Inc.).

### Confocal microscopy imaging

Cells (5 × 10^4^ cells) were cultured onto poly-l-lysin-coated round coverslips for 24 h. Coverslips were then fixed with 4% PFA for 10 min at RT. Cell spots were then blocked with 50 μL of PBS (incl. 2% FBS and 2 mM EDTA) for 10 min before adding anti-human CD33-FITC (1/200, eBioscience, HIM3-4) and anti-human TLR2-PE (1/50, BioLegend, clone QA16A01) antibodies for 2 h at RT. Cell spots were then washed in PBS and analyzed on a Zeiss LSM 700 confocal microcope.

### Monocyte-derived dendritic cells and macrophages

Peripheral blood mononuclear cells were isolated from buffy coats via standard Ficoll centrifugation [Ficoll-Paque PLUS (GE Healthcare)]. To generate monocyte-derived dendritic cells (moDCs) or macrophages, CD14^+^ cells were isolated from PBMCs using the human Monocyte Isolation Kit II (Miltenyi Biotec, Bergisch-Gladbach, Germany). The cells were suspended in RPMI 1640 (Gibco) with 10% FCS and penicillin and streptomycin. CD14^+^ monocytes (1 × 10^6^ cells/mL) were supplemented with 100 ng/mL GM-CSF and 50 ng/mL IL-4 to generate moDCs. For the generation of macrophages, 100 ng/mL GM-CSF alone was supplemented. The suspension was incubated for 3 days at 37 °C and 5% CO_2_. The medium was renewed together with the named cytokines, respectively. At day 6, the cells were adjusted to 1 × 10^6^ cells/mL. Macrophages or moDCs cultured in medium only acted as negative controls. Some wells were pretreated with either 10 µg/mL anti-hTLR2 or anti-IgA2-control antibody for 45 min prior to the addition of IL-26. The stimulants LPS (100 ng/mL), LL37 (10 µM) and IL-26 (2 µM) were added for 24 h.

### Enzyme linked immunoassay

Cytokine levels were identified with TNF-α or IL-6 DuoSet ELISA (R&D Systems).

### Statistics

All analyses were done using GraphPad Prism Version 5.03 (GraphPad Software, Inc.). The “n” in the figure legends always refers to the number of independently performed experiments and can thus be considered as biological replicates. Mann–Whitney *U* test was used to analyze the diseases and healthy controls. For experiments involving cells, the Wilcoxon matched-pairs signed rank test was applied. Statistical significances are depicted as follows; * equals P ≤ 0.05, ** equals P ≤ 0.01 and *** equals P ≤ 0.001.

## Supplementary information

Supplementary Information.

## Data Availability

No datasets were generated or analyzed during the current study.
