# Supplementary material for: Interleukin-26 activates macrophages and facilitates killing of Mycobacterium tuberculosis
Source: Sci Rep. 2020 Oct 14;10:17178. doi: 10.1038/s41598-020-73989-y (PMC7558018; doi:10.1038/s41598-020-73989-y)
Supplement: Supplementary file 1 — Supplementary Information. [file 41598_2020_73989_MOESM1_ESM.pdf]

Supplemental information

Interleukin-26 activates macrophages and facilitates killing of *Mycobacterium tuberculosis*

Heike C. Hawerkamp[1], Lasse van Geelen[2], Jan Korte[2], Jeremy Di Domizio[3], Marc Swidergall[4], Afaque A. Momin[5], Francisco J. Guzmán-Vega[5], Stefan T. Arold[5], Joachim Ernst[4], Michel Gilliet[3], Rainer Kalscheuer[2], Bernhard Homey[1], Stephan Meller[1]

1 Department of Dermatology, Medical Faculty, Heinrich-Heine-University Düsseldorf, Germany
2 Department of Pharmaceutical Biology and Biotechnology, Heinrich-Heine-University, Düsseldorf, Germany
3 Department of Dermatology, University Hospital CHUV, Lausanne, Switzerland
4 Department of Biology, Molecular Mycology, Heinrich-Heine-University, Düsseldorf, Germany
5 King Abdullah University of Science and Technology (KAUST), Computational Bioscience Research Center (CBRC), Division of Biological and Environmental Sciences and Engineering (BESE), Thuwal, Saudi Arabia

Supplemental figures

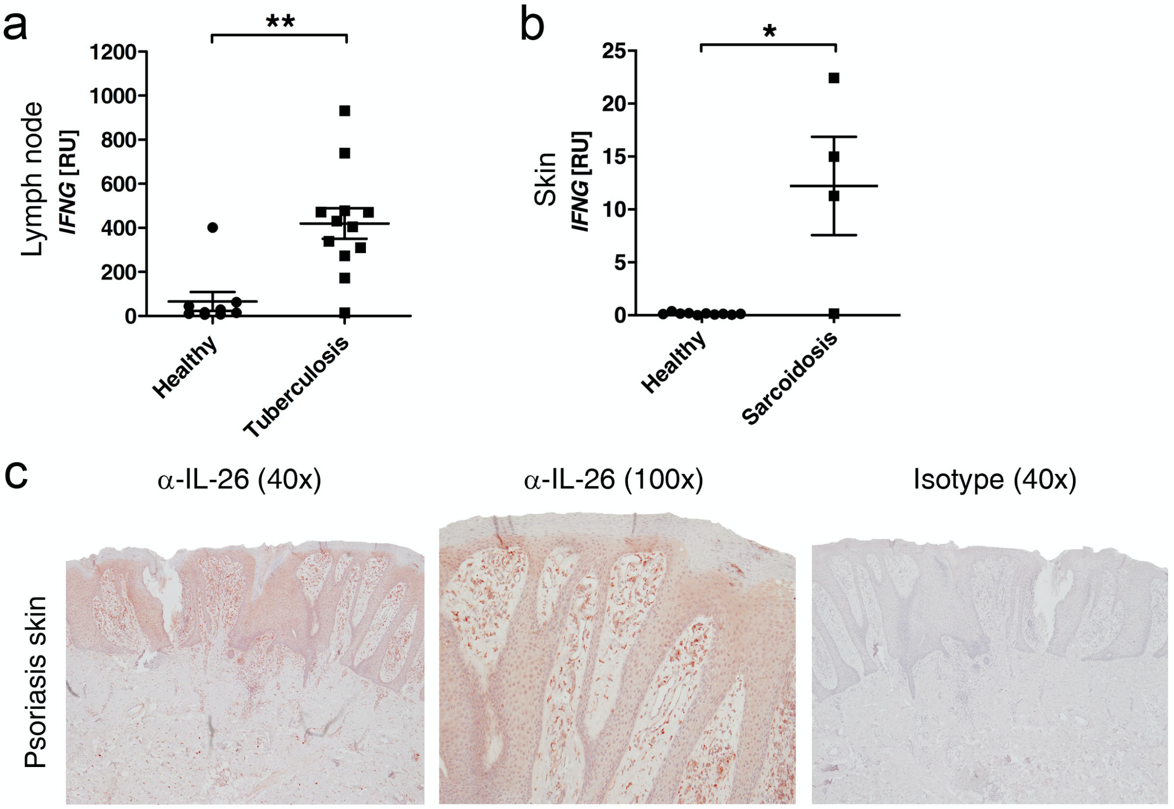

Supplemental Figure 1: ***IFNG* is over-expressed in both tuberculosis and sarcoidosis compared to healthy controls.** qPCR analysis of gene expression of *IFNG* in tuberculosis LN (a, n=12) compared to healthy control LN (n=9) in RNA from FFPE lymph nodes and Sarcoidosis skin punch biopsies (b, n=4) compared to healthy skin controls (n=10). Immunohistochemistry with anti-IL-26 and isotype control on sections (4 µm) from skin punch biopsies from one psoriasis patient (c). Magnification: 40 × for left and right panel, 100 × for middle panel.

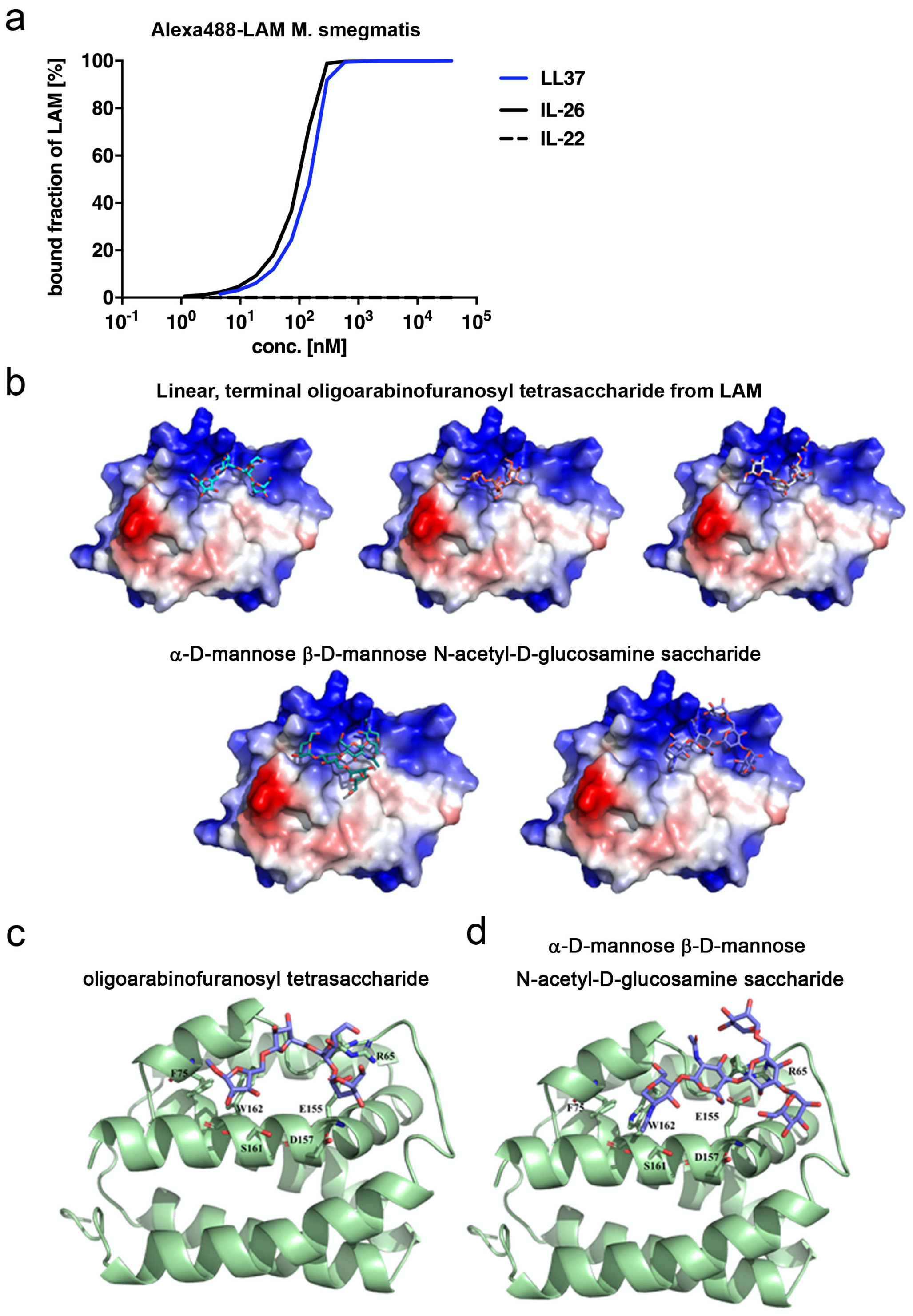

Supplemental Figure 2: (a) Binding of IL-26 to mycobacterial LAM from M. smegmatis was investigated using microscale thermophoresis (MST). The percentages of bound fractions of LAM to different IL-26, IL-22 and LL37 concentrations are displayed graphically. (b) Upper panels; Best docked poses of IL-26 model represented in surface electrostatics with linear, terminal oligoarabinofuranosyl tetrasaccharide from LAM shown in sticks and lower panels; Best docked poses of IL-26 model represented in surface electrostatics with α-D-mannose β-D-mannose N-acetyl-D-glucosamine saccharide. (c) The best scored docked pose of IL-26 model represented in cartoon with linear, terminal oligoarabinofuranosyl tetrasaccharide from LAM represented in sticks and (d) best scored docked pose of IL-26 model represented in cartoon with α-D-mannose β-D-mannose N-acetyl-D-glucosamine saccharide. The interaction residues are labeled appropriately.

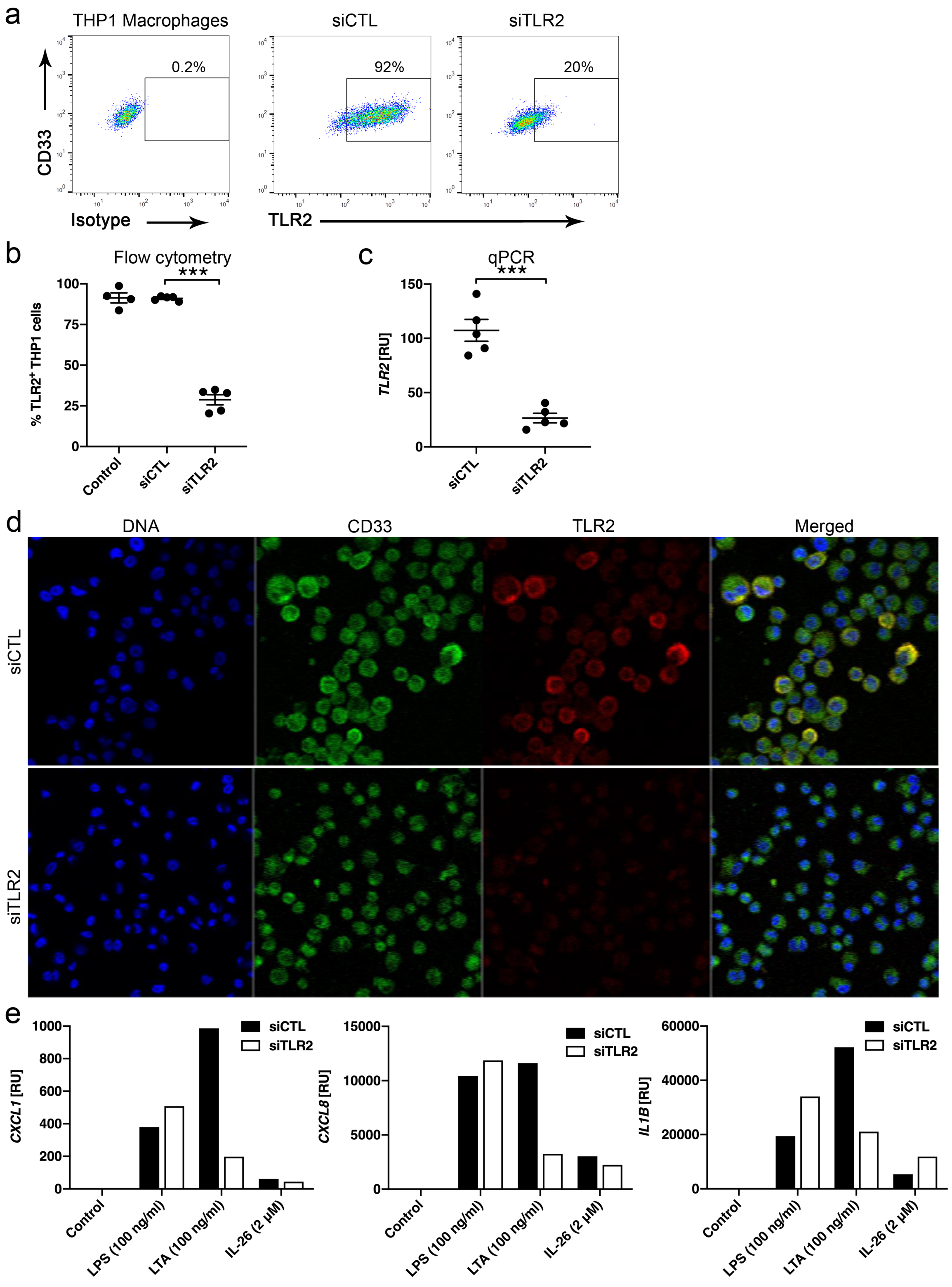

Supplemental Figure 3: **Efficient TLR2 knockdown in THP1 macrophages.** (a) TLR2 surface expression is drastically reduced in siTLR2-treated THP1 macrophages as shown in a representative flow cytometry blot. Both TLR2 protein (b, n = 4 - 5) and TLR2 gene expression (c, n = 5) is inhibited by treated with siTLR2. (d) The reduction in TLR2 surface expression is also visualized using confocal microscopy. (e) TLR2 knockdown leads to strong decrease in *CXCL1*, *CXCL8* and *IL1B* gene expression in THP1 macrophages detected via qPCR (n=1). qPCR-values are shown as relative units compared to 18S RNA. Data are presented as mean + SEM. Statistical analysis was done using Mann Whitney *U* test (*** equals p<0.001).
